# Anti-Infective and Toxicity Properties of Carbon Based Materials: Graphene and Functionalized Carbon Nanotubes

**DOI:** 10.3390/microorganisms10122439

**Published:** 2022-12-09

**Authors:** Naghmeh Hadidi, Maryam Mohebbi

**Affiliations:** Department of Clinical Research and EM Microscope, Pasteur Institute of Iran (PII), Tehran P.O. Box 1316943551, Iran

**Keywords:** carbon-based materials, carbon nanotubes, graphene, antimicrobial, toxicity

## Abstract

Recently, antimicrobial activities of various carbon-based nanomaterials against specific pathogens have become one of the most significant research interests in this field. Carbon nanotubes (CNTs) are promising multidisciplinary nanostructures in biomedicine, drug delivery, genetic engineering, biosensors, and artificial implants. However, the biomedical administration of CNTs is dependent on their solubility, toxicity, and biocompatibility, as well as novel drug-delivery applications through optimization of the drug’s loading capacity, cellular absorption, and continuous release within the target cell. The usage of CNTs and Graphene materials as antimicrobial agents and nanocarriers for antibiotics delivery would possibly improve their bioavailability and facilitate better anti-infective therapy. However, it is worth mentioning that CNTs’ antimicrobial activity and toxicity are highly dependent on their preparation and synthesis method. Various types of research have confirmed that diameter, length, residual catalyst, metal content, surface coating, electronic structure, and dispersibility would affect CNTs’ toxicity toward bacteria and human cells. In this review article, a general study was performed on the antimicrobial properties of carbon-based nanomaterials, as well as their toxicity and applications in confronting different microorganisms. This study could be useful for researchers who are looking for new and effective drug delivery methods in the field of microbial resistance.

## 1. Introduction

Carbon nanotubes (CNTs) were first discovered in 1991 by Ijimia [1,2,3,4,5,6,7]. Ijimia described multi-walled carbon nanotubes (MWCNTs) in carbon ash obtained during C60 fabrication in the arc evaporation method. Nowadays, the synthesis of CNTs is performed via arc discharge, laser ablation, chemical vapor deposition (CVD), ball milling, or flame procedures. The selection of the synthesis method mostly depends on desired properties required for a particular application. Centrifugation, oxidation, filtration, and acidic treatment are complementary steps being used for CNTs’ purification. The SWCNTs produced by arc discharge are of high purity and quality. CVD-produced CNT characteristics including diameter are directly affected by operation pressure, temperature, hydrocarbon source and concentration, and reaction type time, and CVD is categorized as CCVD (catalytic chemical vapor deposition), microwave plasma (MPECVD), and oxygen-assisted CVD.

Commonly used metal catalysts are Ni, Co, Fe, or their combinations, regarding the carbon source hydrocarbons such as methane, acetylene, ethane, ethylene, or their mixtures are favorable. The flame synthesis method is a low-cost large-scale method that can produce carbon nanotubes with customized surface properties with three main constituents: Metallic catalyst particles, a heat source, and a source of carbon [7].

CNTs are generally categorized according to the number of graphene layers as single-wall carbon nanotubes (SWCNTs) or multi-wall carbon nanotubes (MWCNTs). SWCNTs are made from one rolled layer of graphene with a 1–2 nm diameter, and MWCNTs are made from more than two rolled layers of graphene with diameters of up to 100 nm [1,2,3,4,5,6,7]. Table 1 compares SWNTs and MWNTs in different aspects [7]. CNTs can exist in three unique geometries including armchair, zig-zag, and chiral. CNTs’ chirality directly influences their mechanical, electrical, and optical properties, as well as their application in different fields [7].

CNTs are promising nanostructure candidates for biomedical applications, and pharmaceutical nanotechnology CNTs are comprehensively described in the literature as nanomaterials with unique physicochemical properties such as a high-surface-area-to-volume ratio, optical, thermal, mechanical, and electrical properties, functionalization possibility, and high loading capacity for biomolecules and genetic components. CNTs are functionalized to deliver one or more hydrophobic, hydrophilic, and genetic materials simultaneously. Interestingly, the nano-needle shape of CNTs can protect genetic material from enzymatic digestion and enhance their permeability from biological barriers and cell membranes [1,2,3,4,5,6,7].

CNTs are promising multidisciplinary nanostructures in biomedicine, drug delivery, genetic engineering, biosensors, and artificial implants. However, CNTs’ solubility, toxicity, and biocompatibility are important milestones and restrictions in their biomedical administration. CNTs are insoluble in most solvents. To overcome such problems, CNTs can be functionalized with biocompatible polymers and surfactants by covalent and non-covalent functionalization methods. Surface modification and polymeric functionalization with phospholipid PEG derivatives and surfactants would markedly improve CNTs’ aqueous dispersion and biocompatibility. Moreover, functionalization would also facilitate secondary conjugation with drug molecules. In addition, modern forms of drug delivery are correlated with the optimization of the drug’s loading capacity, cellular absorption of drug carriers, and continuous release of the drug within the target cell. Thus, surface modifications, similar to the ones developed in natural cells, could be initiated by biomimetic production techniques or methods [8].

Functionalized CNTs are not intrinsically immunogenic but are capable of activating immune system cells including monocytes, macrophages, and DCs after cellular reuptake. The application of SWCNTs is proposed as immune stimulator candidates and antigen carriers in vaccine studies [4], while nano-suspensions, as the main type of nanofluids, are highly bio-medically functional in terms of drug delivery, medical treatment, disease diagnosis, anti-bacterial uses, wound dressing, and freezing [9].

CNTs are widely used nowadays for cancer treatment as a supplement for carbon nanotubes and irradiation, mixed-drug treatment, a DNA delivery vector for gene therapy, the purposeful delivery of siRNAs, and the design of a CNT-based array biosensor using particular types of antibodies. CNTs’ particular features including the large surface area, conjugation, and capsulation potential, as well as their targeting capacity, have turned CNTs into an important factor for improving the speed, efficacy, and satisfactory selection of proper treatment and diagnosis of cancer [10].

## 2. Overview and Applications of Carbon-Based Nanomaterials as Antimicrobials

### 2.1. Antimicrobial Activities CNTs

The antimicrobial properties of CNTs mostly depend on their composition, surface modification, specific microorganisms, and surrounding environment. Most of the possible antimicrobial mechanisms of CNTs are based on the invasion of the microorganism cell wall and the induction of structural damage. Oxidative stress induction via the production of toxic materials and reactive oxygen species (ROS) in which electrons are removed from the microbial surface and cell death would occur [11]. Some researchers confirm that when CNT size decreases, their surface-to-volume ratio increases and ends in stronger interaction with the microorganism cell membrane. They explain that disruption of the cell membrane, metabolic procedure, and morphology, as well as the enhanced efflux of plasmid DNA, RNA, and cytoplasmic materials, are the main mechanisms of action of CNTs’ bacteriostatic properties [11,12,13,14,15,16,17,18]. The use of CNTs as novel drug-delivery systems for antibiotics will also increase their bioavailability and facilitate targeted therapy. Kang et al. first announced the size-dependent antibacterial properties of SWNT against *E. coli* in 2007. Their complementary studies declare that SWCNTs (single-wall carbon nanotubes) are more toxic to microorganisms and Gram-negative and Gram-positive bacteria in comparison to MWCNTs (multi-wall carbon nanotubes) [11,19,20,21,22]. Better penetration into the cell wall would occur for CNTs with a smaller diameter.

**Figure 1:**
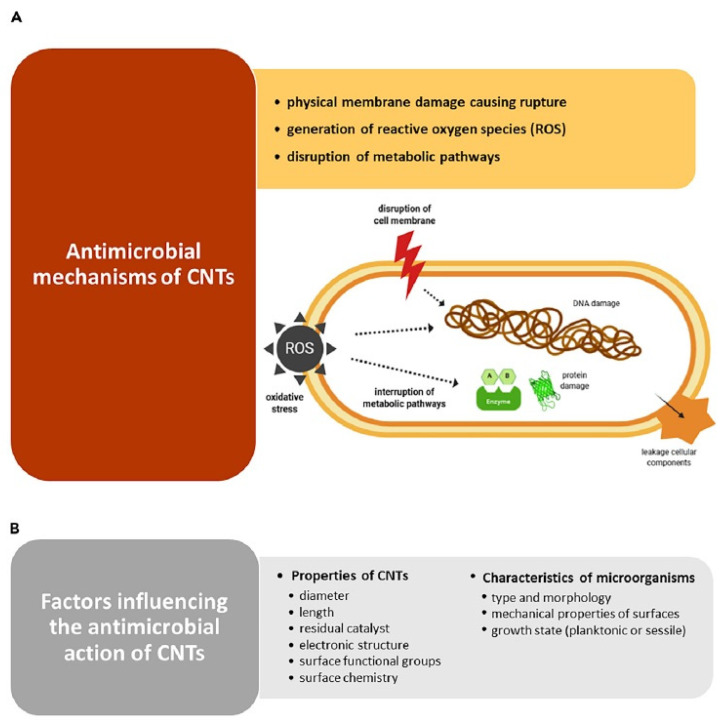
Mechanism of anti-microbial activity of carbon nanomaterial. Adopted from [12].

Table 2 summarizes the different antimicrobial activities of carbon nanomaterial against specific pathogens, and Figure 1 describes the possible mechanism of the antimicrobial activity of carbon nanostructures [12]. Bing et al. evaluated the effect of CNTs’ negative and positive surface charge on bacterial death. The generation of reactive oxygen species (ROS), such as hydroxyl radicals, was disclosed as a responsible factor for the deterrence of bacterial growth and cell death [11,23,24,25,26].

Chen et al., Rodrigues et al., and Liu et al. reported that the antibacterial activity of CNTs may also depend on microorganism properties including type and morphology, the mechanical properties of cell surfaces, and the growth state [12,27,28,29]. Chen et al. declare the hypothesis of ‘‘nano-darts’’ as the main cause of bacteria death [27]. Gram-positive bacteria including *Staphylococcus aureus* and *Bacillus subtilis* are more susceptible to single-wall carbon nanotubes due to their spherical shape and membrane softness [15,26]. Biofilms, free-floating, and rod-shaped cells are more resistant to the bactericidal activities of CNTs [29]. CNTs are chemically stable cargoes for the delivery of therapeutic molecules including antibiotics and antimicrobials. CNTs loaded with antibiotics would be a promising strategy to combat antibacterial resistance Moreover, due to their intrinsic antimicrobial activity, the emergence of drug-resistant strains has added to the significance of studies being undertaken on carbon nanotubes. CNT’s’ exclusive features in improving the efficiency of drugs such as antibiotics, reducing drug dosage, and antibiotic resistance have been studied in the destruction of *Acinetobacter bumanii* and the obtained results proved to be satisfactory [30].

In addition, MWCNT nanofluids as compounds with prevalent antibiotics such as Kanamycin and Streptomycin are effective in displaying superior characteristics including increased penetration into the bacterial membrane, heightened efficiency in lower concentrations compared to prevalent treatment dosages, and lower bacterial resistance to antibiotics in the treatment of *M. fortuitum* [31].

Treatment of the resistant strain of *Klebsiella pneumoniae* using the +f-MCWNTs antibiotic is sufficient for proving the antibiotic efficiency in lower dosages, reducing antibiotic resistance, and increasing the permeability of the cell wall toward the antibiotic due to the presence of MWCNTs [32].

They would be new options for the production of medical devices and prosthetic implants [12,33,34,35,36,37]. Malek et al. showed that silicone materials decorated with aligned multi-wall carbon nanotubes can reduce the possibility of biofilm formation by up to 60% and might be suggested as new material for medical device manufacturing [38]. Vagos et al. also demonstrated that a polydimethylsiloxane (PDMS) matrix containing 10% pure MWNTs was effective in the 20% reduction of *E. coli* adherence in simulated conditions to the urinary tract and offered this material for urinary tract medical devices [39].

### 2.2. Antimicrobial Activities of Functionalized CNTs [15]

Despite the promising potential of CNTs in biomedicine, the hydrophobic structure and innate toxicity of pristine and pure single-wall and multi-wall carbon nanotubes might be a drawback. However, surface functionalization would increase interaction with the cellular membrane and antimicrobial activity of CNTs. This would normally happen as a result of better aqueous dispersion, improved biocompatibility, and reduced toxicity for human cells [12]. It seems that functionalized MWCNTs with amine, carboxyl, nitrogen ions, and ethanolamine show good antibacterial properties against *E. coli* and *S. aureus* when used in medical devices [40,41,42,43]. Amine-Functionalized MWCNTs were reported to significantly increase *E. coli* and *S. aureus* MIC in comparison to PCL (poly ε-caprolactone) [41]. MWCNTs being functionalized using acylation reactions, supplemented by the use of INH medicine to obtain a proper dosage of the nano-medicine and heighten the efficiency and lower antibiotic resistance, have been proven effective in the treatment of *Tuberculosis* [44].

Similar results were reported by Zardini et al. [42]. Another study was conducted to investigate the level of pro-inflammatory cytokines in macrophages derived from THP1 and A549 cell strains contaminated by *Klebsiella pneumoniae* as the resistant strain, which was treated by f-MWCNTs+cip. The results of this study illustrated that exposure to ciprofloxacin has been highly influential in increasing cytokines, both at transcription and translation levels. In contrast, +f-MWCNTs ciprofloxacin was effective in the expression and secretion of cytokines in macrophages derived from contaminated THP1 [45].

Numerous challenges have been reported in counteracting nosocomial infections. Carbon nanotubes, as functionalized nanofluids, are considered an appropriate approach for the treatment of this category of infections. The simultaneous prescription of functionalized carbon nanotubes as well as meropenem, in a nanofluid environment, was significantly effective in reducing the growth of the *Pseudomonas aeruginosa* strain. Moreover, through heightening drug stability, the carbon nanotubes were effective in reducing *Pseudomonas aeruginosa* antibiotic resistance in lower dilutions compared to antibiotics [46].

The effectiveness of MWCNT nanofluids, being functionalized with a carboxylic acid, is vastly different from the effectiveness of non-functionalized multi-walled carbon nanotubes. It seems that this function has been modified after bacteria’s exposure to the nanofluid and, possibly, the lower bacterial growth rate can be attributed to the connection between functionalized MWCNT nanofluids and the bacterial membrane. This has resulted in the destruction of the membrane’s integrity and heightened antibiotic efficiency. Thus, functionalized MWCNTs would have antimicrobial impacts on *Staphylococcus aureus* and would overcome the antibiotic resistance of this strain [47].

In another study, different modes of drugs were examined as free-standing medicine, as functionalized MWCNTs, non-functionalized MWCNTs, a drug in combination with a non-functionalized MWCNT nanofluid, and as a drug in combination with a functionalized MWCNT nanofluid on the *Klebsiella pneumoniae* strain. The results suggest that the drug in combination with the functionalized MWCNT nanofluid was highly effective in inhibiting bacterial growth [48].

Highlighting the antimicrobial activity of amine-MWCNTs was associated with stronger interaction between the cationic nature of amine-MWCNT and negatively charged cells of bacteria and extensive cell membrane lysis, which led to its bactericidal effect. These outcomes suggested functionalized carbon nanotubes as novel nano-antimicrobial materials for the construction of medical devices and implants [12].

### 2.3. Functionalized CNTs as the Carriers for Antibiotics’ Delivery [12]

Carbon nanotubes are proposed as promising materials in the battle against antimicrobial drug resistance (AMR). MWCNT nanofluid conjugated with Isoniazid and Fluoxetine with a nano-drug delivery system is highly effective in treating infections as well as reducing drug resistance in *Mycobacterium Tuberculosis* clinical strains [49]. Covalent conjugation of cephalexin with PEGylated MWCNTs improved the bactericidal activity of cephalexin against Gram-negative and Gram-positive bacteria simply by their anti-adhesive characteristic [50]. Azithromycin conjugated with SWCNTs was also reported to show higher antibacterial activity against *Micrococcus luteus* [51]. Titanium discs coated with Rifampicin-MWCNTs showed better inhibition for the formation of a *Staphylococcus epidermis* biofilm [52].

Antimicrobial photodynamic therapy (APT) is a non-antibiotic agent for bacterial contamination. This idea was well supported by research performed on an NIR application along with carbon nanotubes conjugated with photosensitizers including porphyrin and DTTC (3,3′ –diethylthiatricarbocanine fluorophores) [53,54]. The results support the idea that APT would help kill *Pseudomonas aeruginosa* by increasing the temperature after laser irradiation [53,54].

CNTs conjugated with Antimicrobial peptides (AMP) including EP (epsilon-polylysine), PLL (polyelectrolytes poly (l-lysine), PGA (poly (L-glutamic acid), and Nisin were also investigated for application in medical devices. High antibacterial activities against *E coli, P. aeruginosa,* and *Staphylococcus epidermis* were also reported [55,56,57].

Cell lysis by lytic enzymes such as LSZ (lysozyme), Lysostaphin, and Laccase have been proposed as promising alternative antimicrobials specially designed to combat methicillin-resistant *S. aureus*, biofilm formation, and anti-sporicidal activity of *B. cereus/B. anthracis* [58,59,60].

The application of bio-nanofilms in medical devices and implants, which are generally silver or metal-coated CNT-based films, was found to be effective against a broad range of bacteria through reduced bacterial adhesion [12]. Among different Bio-nanofilms, AgNPs (silver nanoparticles), AgNPs-DNA (silver nanoparticles stabilized with DNA), ZnHa (zinc hydroxyapatite), and PdNPs (palladium nanoparticles) were the most evaluated CNT-metal conjugates for antibacterial activity against *E. coli, A. aureus, B subtilis, P aeruginosa, S. epidermis,* and *K. pneumonia* [61,62,63,64,65,66,67].

Polymers have been widely applied to develop CNT Nanocomposites with improved structural, mechanical, biocompatibility, biological stability, and cost effectiveness [12,28,68]. Among various polymeric CNT composites, PEG (polyethylene glycol), PLGA (poly(lactic-co-glycolic acid), PEI (Poly ethyleneimine), and polypyrrole composites were found to achieve significant inactivation of a broad spectrum of Gram-negative and Gram-positive bacteria, including *E. coli* and *S. epidermis,* up to 98% when used as a wound dressing or in medical devices [37,68,69,70,71].

Chitosan-MWCNT nanocomposites have been enormously explored as probable antimicrobial surfaces for wide implementation in biomedical applications including wound dressing, tissue engineering, biosensing, and drug delivery [72]. It has been stated that the incorporation of MWCNTs would be an added value that intensifies the innate antibacterial/antifungal activity of chitosan. Carboxymethyl chitosan (CMCS), aminohydrazide, and aminosalicyl-hydrazid-cross-linked chitosan are some examples of chemically modified chitosan exploited with functionalized MWCNTs to achieve stronger antimicrobial activity against Gram-positive bacteria [34,35,73]. Shi et al. and Pramanik et al. [70,74] studied the antimicrobial and anti-adhesive properties of functionalized-MWCNT/hyperbranched poly(ester amide) (HBPEA) thin films. The higher the loading of MWCNTs, the less cell adhesion was observed to this functionalized MWCNT-HBPEA, and specific Gram-positive antibacterial activity was achieved. PEG-functionalized CNTs and thermoplastic polyurethane (TPU)-PEG electro-spun Nanofibers were highlighted as promising CNT/polymer biomaterials with less possibility of auto aggregation [70]. Anti-adhesive properties of CNT/polymer Nanocomposites are partially related to the surface smoothness and uniformity of the polymer coating as well as π–π interactions [70,74,75,76,77,78].

Polymeric surfactants are another tool in the preparation of stable dispersed CNTs. Researchers declare that the antibacterial activity of single-walled carbon nanotubes (SWCNTs) is dispersed in surfactant solutions. Sodium cholate showed the weakest antibacterial activity against *S. enterica*, *E. coli*, and Enterococcus faecium in comparison to sodium dodecylbenzene sulfonate and sodium dodecyl sulfate. It was reported that increasing nanotube concentrations up to 1.5 mg/mL will potentiate the antibacterial activity of CNTs as an effective alternative to antibiotics, especially regarding multidrug-resistant bacterial strains [13].

It could be concluded that metals, antimicrobial agents, and polymers play key roles in the antibacterial and AMR properties of CNT-based compounds. The synergistic effect of CNT-based antimicrobials was suggested to be caused by the inhibition of the cell wall, inhibition of protein synthesis, increase in cell membrane permeability, loss of membrane integrity and potential, protein dysfunction, oxidative stress, promotion of microorganism cell wall contact, and change in surface hydrophobicity and roughness as a bacterial anti-adhesion strategy [12,55,56,57].

### 2.4. Antimicrobial Activities of Graphene [14]

Graphene materials (GMs) are one of the new carbon-based, alternative strategies for using materials with inherent antibacterial properties to prevent infection. Recent research has suggested that the antibacterial activity of graphene and graphene-derived materials occurs after direct physical and chemical interactions between GMs and bacteria, which cause the lethal degradation of cellular components, mainly proteins, nucleic acids, and lipids. GMs tend to accumulate in membrane proteoglycans, leading to membrane damage. GMs interrupt the replication phase by interacting with the hydrogen groups of the RNA/DNA of bacteria. They can also indirectly determine bacterial death after entering the physiological environment by activating the inflammatory pathway caused by active species [14].

The analysis results suggest that the graphene sheet is capable of automatically penetrating the dimer protein. The penetration of graphene sheets into the protein–protein interface would destabilize the Protein–Protein Interaction (PPI) by disturbing the hydrophobic interactions resulting in the decomposition of the protein complex [79].

Graphene is a carbon layer of the graphite structure, composed of hybridized carbon atoms linked by longitudinal bonds tightly packed into a honeycomb lattice to form a two-dimensional crystal [80,81]. Graphene derivatives include graphene oxide (GO) and reduced graphene oxide (rGO), which are created by chemical modifications in graphene to improve its properties and can be used in various fields [82]. GO have oxygen functional groups including hydroxyl, carboxyl, carbonyl, and epoxy which are mostly obtained from an oxidized graphene molecule and graphite acid oxidation. GO is insoluble in organic solvents such as alcohol, and toluene due to its strong hydrophilic properties. Furthermore, it is remarkably effective in force harvesting and electronic applications [82,83]. The GO structure includes many functional groups, which are capable of covalently binding to biological molecules and growth factors to strengthen cellular proliferation and differentiation. It can be deciphered that hydrophilic surfaces such as GO would be easily proliferated; however, upon regulating the rGO’s (hydrophilic) oxygen level and the use of appropriate additives, an efficient material for TE and medical purposes would be developed [84].

rGO is obtained by chemical or thermal reduction of oxygen in functional groups in GO material. In addition, rGO has a wide surface area and strength, high reactivity, and biocompatibility [85,86]. The antimicrobial properties of GMs depend largely on their lateral size, the number of layers, particle shape, surface modifications, agglomeration, and dispersion. Lateral size is a determining factor in the antimicrobial effectiveness of GMs. Research has confirmed that the larger the GM lateral size, the stronger the absorption capacity attributed to the higher surface energies.

According to the conducted research, the number of graphene layers has a major influence on its antimicrobial activity. Increasing the number of layers of GMs could enhance thickness and diminish dispersion. Moreover, the number of layers of GMs could increase the tendency to aggregate, leading to less contact between GMs and microorganisms. Generally, the number of layers influences the surface features that induce the basal plane’s antimicrobial activity, which shows both the edges and surface of GMs are important factors in antimicrobial activity. An example of this is the study carried out by Mangadlao, which revealed that an increased number of GO sheets have a stronger antimicrobial effect against *E. coli* [87,88].

The particle shape considerably influences the antimicrobial activity of nanoparticles. Studies have shown that nanoparticle shapes are essential for their interaction with the lipid bilayer in a translocation process. Additionally, the easy permeation of graphene nanoparticles into the cell membrane owing to the low energy barrier of these sharp-corner protruded particles can be caused by antimicrobial activity [89,90]. Akhavan and Ghaderi et al. reported that the sharp edges of graphene oxide nanowalls (GONWs) and graphene oxide nanowalls (RGNWs) significantly reduced the rate of survival of both *E. coli* and *Staphylococcus aureus* (*S. aureus*) [91].

The interaction between GMs and other molecules, such as proteins, lipids, DNA/RNA, and other materials is crucial for antimicrobial activity. The tendency of intact graphene to agglomerate potentially reduces its contact with other particles [88]. Surface modifications of graphene through covalent and noncovalent modulation have been found to play an important role in preventing particle agglomeration and, as a result, affect their antimicrobial activities [87,92,93]. Recent research has suggested that rGO has stronger antimicrobial activity than GO against *S. aureus* and *E. coli* [94]. According to other reports, rGO can inhibit the proliferation of *E. coli*, while no cytotoxicity has been observed in the case of GO [95].

Research has suggested that the antimicrobial effect of GMs may be increased by the effect of covalent modulation with oxygen-containing groups. Oxygen groups can influence the GMs’ amphipathic and chelating effect of the generators, which subsequently alters their antimicrobial activities [96,97]. Consequently, GMs can affect the survival of microorganisms through adsorption interactions between GMs and molecules, ions, and other substances [14,88].

GMs, due to the high surface energies. are susceptible to agglomeration that modulates the edge and surface characteristics of the nanoparticles and changes their antimicrobial activities. In the case of CNTs, one of the main factors that drive their antimicrobial activity is the tendency to aggregate, which reduces the surface area and changes the shape of the nanomaterials [98]. The density of GMs weakens their dispersibility and absorption, which changes the efficiency of the blades and thus reduces their interaction with microorganisms. It has been reported that rGO is stronger than GO in bacterial inactivation. This is attributed to the entrapment of *E. coli* and its ability to gradually cover the bacteria during the formation of rGO beads in suspension [88,99].

Different experimental conditions should be considered when evaluating the antimicrobial activities of GMs. Experimental conditions such as the state of the material applied, the type of bacteria (aerobic and anaerobic), the medium applied (in vitro and in vivo), and the genus of microorganisms such as the shape (rod and round) and class (Gram-positive and -negative). Controlling the growth of microorganisms is very important because each microorganism has its own capacity in physicochemical conditions. It has been reported that the antimicrobial activity of the rough surface of graphene layers is stronger against *P. aeruginosa* than against *S. aureus*. This phenomenon was interpreted as an indicator of the antimicrobial effect degree and is highly dependent on the selected bacterial species [88,100].

Summarizing GMs’ advantages, conductivity, mechanical properties, antibacterial properties, detection, and water decontamination are worth mentioning. GMs possess wide-ranging antibacterial and antiviral applications. GMs could be used for industrial water treatment to delete ions, bacteria, and other contaminants [101,102,103,104,105]. GMs’ activity is not targeted toward specific receptors or pathways, so resistance could be developed by bacteria after long exposure, which is the disadvantage of GMs application [14].

### 2.5. Effect of CNTs Preparation Methods on Their Antimicrobial Activity, Toxicity, and Mechanism Insight [18]

CNTs toxicity to human cells is a major concern that should be addressed carefully while focusing on its antimicrobial properties. Various kinds of research confirm that diameter, length, residual catalyst, metal content, surface coating, electronic structure, and dispersibility affects CNTs toxicity in bacteria and human cells [18]. The results of animal research suggest that long-time exposure to CNT would result in permanent inflammation, lung cancer, fibrosis, and the destruction of genes within the lung. The presence of MWCNTs within the human body would result in the production of cytokines such as TNF- α and IL-1 β from the immune cells involved in the development of toxicity. Moreover, SWCNTs would result in acute effects including inflammation, granuloma synthesis, collagen deposition, fibrosis, and genotoxicity within human lungs; however, the use of novel methods such as functionalization would assist researchers in the development of nanotubes with higher length, width, and curvature values, though with lower toxicity [106].

CNTs that are prepared by Arc discharge, electrolysis, laser ablation, chemical vapor deposition (CVD), and sono chemical/hydrothermal methods are suitable as electrochemical biosensors and antimicrobials. The synthesis method and extra modifying additives are critical parameters in CNTs’ applications. For example, carbon-nanotube array-based microfluidic devices and Molybdenum disulfide-MWCNTs (MoS_2_-MWCNTs) are CNT-based biosensors with improved selectivity due to the negatively charged carboxyl group on MWCNTs for virus identification and chloramphenicol/dopamine detection, respectively [18,107,108,109]. In another study, the impacts of Bromocriptine (BRC)-conjugated MWCNTs on lung cancer cells (i.e., A549 and QU-DB) and MRC5 have been studied using MTT and Flow Cytometry tests. The results of this study suggest that this nano-medicine has a significant lethal effect on cancer cells; however, no toxicity effect has been observed on MRC5. In addition, nano-medicine is significantly capable of inducing apoptosis in lung cancer cells, as compared to simple medicines [110].

The beneficial aspect of CNTs might seem a revolutionary strategy against increasing microbial infections in clinics and hospitals caused by ignorant usage of antimicrobial agents.

CNTs’ antimicrobial potency has attracted attention and interest in the usage of CNTs as coatings or dressings in medical devices and hospital settings to prevent nosocomial infections [18,58,111,112,113,114]. Membrane damage, ROS activation, suppressed metabolic activity oxidative stress, extraction of phospholipids, and DNA/RNA release are considered the main mechanisms for insight into CNTs’ antibacterial activity [18].

Functionalized CNTs with strong oxidizing groups will significantly improve their aqueous dispersivity for biotechnological applications [18,115]. Aggregation and dispersivity properties might be considered. Short-length SWCNTs show higher bactericidal activity due to the higher self-aggregation possibility [18]. Smaller diameters cause more damage to the cell membrane through more cell–surface interactions. Meanwhile, the presence of amorphous carbon species as impurities and carboxyl groups on CNTs’ surface directly affects CNTs’ toxicity and antibacterial activity. Therefore, highly purified, short-length, small-diameter, functionalized CNTs could be considered unique selective bactericidal agents [27,116,117,118,119].

Carbon nanomaterials are nanostructures containing impurities based on the applied synthesis, preparation, and purification methods. Metallic, nanographitic, and amorphous carbon-based impurities are the commonly found impurities in CNTs. Pumera et al. impressively explained how such impurities are capable of dramatically influencing redox properties as one of the mechanisms involved in their antimicrobial activity [120].

### 2.6. Carbon Nanotubes as Antimicrobial Agents for Water Disinfection and Pathogen Control [16]

Waterborne diseases considerably influence human health and cause high mortality worldwide. Antibiotics have been known to treat bacterial strains, and their excessive use enhances bacterial resistance. Hence, there is a strong need to find other methods of water disinfection with more efficient microbial control. CNTs have shown strong antimicrobial properties due to their remarkable structure. Among waterborne diseases, typhoid fever, cholera, and dysentery can be mentioned, which significantly affect human health and are the cause of high mortality worldwide. Clean, pathogen-free drinking water is necessary for living organisms. Removing pathogens from contaminated water is an essential requirement for human health and the environment. The process of removing pathogens from water is difficult due to the fluctuating concentration of pathogens and the type of pathogens present in the incoming water. Chlorine, ozone, and chlorine dioxide are common disinfectants that can control microbial growth, but they have short-term reactivity and can be problematic due to the formation of toxic disinfection byproducts. Therefore, it is important to extend an alternative technique that can effectively improve the reliability of disinfection [16,121,122].

Brady et al. developed the first SWCNT filter as a PVDF microporous membrane filter for water disinfection via the removal and inactivation of viruses and bacteria from an aqueous medium. These nanofillers were found specifically effective against *E. coli* and *S. aureus* [18,123,124]. Ali et al. also disclosed surface functionalization and novel nanocomposites made of CNTs, iron oxide, titanium oxide, ferric oxides, and silver nanoparticles as promising agents for disinfection and decontamination of drinking water from *E. coli, S. aureus,* and *P. aeruginosa* [16,18,124,125].

The interaction opportunity of CNTs with bacterial cells and the antibacterial activity of CNTs are increased in higher dispersivity [126]. According to Liu et al., individually dispersed SWNTs in a Tween-20 saline solution have stronger antibacterial activity than SWNT beads. They hypothesized that individually dispersed SWNTs act as multiple mobile “nano-darts” in solution and constantly attack bacterial cells, leading to the disruption of bacterial cell integrity and causing cell death [27]. Polymer conjugation is another strategy to potentiate the antimicrobial activity of CNTs. Molecular weight, chemical composition, surface charge, and functional groups of polymers directly affect the bactericidal properties of CNTs [16,120].

External factors such as CNTs’ dosage, the culture medium, treatment time, and bacterial species are important. In recent research, bactericidal behavior was found to be dependent on incubation time. It has been observed that Gram-positive Bacillus subtilis showed more cell inactivation after longer incubation with SWNTs [123,127].

Lilly et al. also found that SWCNTs and conjugated SWCNT-H_2_O_2_ are both effective in the deactivation of *B. anthracis* spores in comparison to non-treated with MWCNT and/or unconjugated oxidizing agents such as H_2_O_2,_ NaOCL at the same concentration. This phenomenon was explained through the synergistic antimicrobial effect of each component [16,128]. For example, Arias and Yang notified that SWCNTs functionalized with hydroxyl and carboxyl groups exhibited extremely strong antibacterial activity in Gram-positive and Gram-negative species while amine-functionalized SWCNTs were considerably less effective. Steric hindrance and less direct contact caused by the long amine-terminated chain were suggested as the reason for this huge difference in antimicrobial potency [16,129].

From a safety point of view, CNTs’ interaction with biological systems may give rise to allergy, cytotoxicity, DNA destruction, and protein malfunctions [130]. Different levels of toxicity would occur depending on the size, shape, length, diameter, surface coating, surface charges, stability, and dispersivity of CNTS and the tissue type and mode of interaction with human cells. Therefore, toxicity evaluation is very critical for the commercialization of CNTs as novel antimicrobial agents [131,132,133].

### 2.7. Photocatalysis and Titanium Coatings of CNTs [134]

TiO_2_ (Titanium oxide) is one of the most expensive and widely used photocatalysts with bactericidal properties. Researchers tend to design a combination of TiO_2_ and ZnO (Zinc oxide) and semiconductors to achieve high photosensitivity, redox potential, and photocatalytic activity with lower cost and toxicity [134]. Researchers evaluated the step-by-step inactivation of *E. coli* by photocatalysis. They declare that bacterial cell membranes are damaged by the process of photocatalysis caused by oxidative stress. Carré et al. had similar results on the photocatalytic effect of lipids and proteins on the elimination of *E. coli* by photocatalysis. Siddiqi et al. reported that photo-excited ZnO nanoparticles diffusing through the cell wall would inactivate the cytoplasmic protein and carbohydrate via the release of ROS molecules. Takao et al. suggested that the presence of a peptidoglycan layer increases the bactericide effect of photocatalysis. Rodríguez-González et al. also described the existence of lesions in the bacterial cell wall caused by ROS molecules and metal particles [134]. Kerek et al. showed that the photocatalyst coating of graphene with TiO_2_ and ZnO caused a significant (*p* < 0.001) reduction in pathogen numbers compared to the control. It is assumed that photocatalysis and titanium coatings of carbon-based material (CNTs and Graphene) would be a potential alternative to fighting antimicrobial resistance, which has significant bacterial reduction capacity against environmental pathogens [134].

## 3. Conclusions, Challenges, and Prospects

In summary, the present review thoroughly explains the importance of CNTs’ purity, functionalization method, and mechanistic insight into how physicochemical properties determine the specificity, selectivity, and antibacterial potency of CNTs and carbon-based material. It is worth mentioning that better (eco-) toxicological patterns of CNT should be obtained through functionalization and conjugation to minimize the negative impact on human cells. On the other hand, producing suitable cost-effective f-SWCNTs and f-MWCNTs is considered a challenge to successfully compete with low-price conventional antimicrobials. After suitably addressing toxic and economic concerns through green technology and environmentally friendly modification, most of the limitations and restrictions may be bypassed, and CNTs’ vast potential would be revealed in environmental pollution and contamination control where microbial control is essential and highly required.

## Figures and Tables

**Table 1:** Comparison between SWCNTs and MWCNTs. Adopted from [7].

SWNT	MWNT
Single layer graphene	Multiple graphene layers
Synthesis requires catalyst	No catalyst is required
Difficult bulk synthesis due to the requirement of appropriate growth and atmospheric condition.	Easy bulk synthesis
Poor purity	High purity
Greater chances of defects during functionalization	Lesser defect chances but when this occurs, it is hard to recover
Aggregation in the body is less	Aggregation in the body is greater
Easy assessment and characterization	Structure is complicated
More pliable and easily twisted	Twisting is not easy

**Table 2:** Antimicrobial activities of various carbon nanomaterials against specific pathogens. Adopted from [11].

CNMs/ Nanocomposite	Fabrication Procedure	Size (Diameter/Length)	Concentration/ Catalyst	Target Species	Activities	Efficacy (%)	Effect and Mechanism of Action
SWCNTs	- ^a^	<2 nm/5–30 µm	-	*E.coli, S. aureus*	Disinfection activity	38.89	Bacterial adhesion or deposition onto bacterial cell
SWCNTs	-	0.75–1.2 nm	-/Amorphous silica	*E.coli* k12	Antibacterial activity	79.9	Cell membrane damage, efflux of cytoplasmic contents
SWCNTs	Arc discharge	0.7–2.0 nm	20%/Metallic catalysts	*E.coli* K12 TG1	Interaction between bacterial cells and SWCNTS	50	Morphological/mechanical damage in cells, higher oxygen consumption rate, lower bioluminescence intensity of cells
SWCNTs	-	0.83 nm	5 µg/mL/-	*E.coli, B. subtilis*	The collision between bacterial cells and SWCNTS may damage bacterial cells	-	Cell wall damage, leakage of intracellular contents, decreased cell volume and height, enhanced bacterial surface roughness
SWCNTs-PVDF	Vaccum-assisted deposition	1.21 nm/10 to 20 µm	0.3 mg/cm^2^	Natural organic matter, metals, bacteria (*E.coli* K12), viruses	Microporous membrane for removal of rival and bacterial pathogens	79	A fluorescence-based viability kit
SWCNTs-Ag	Solution mixing	<2 nm/5–30 µm	-	*E.coli, S. aureus*	Disinfection activity	70.24, 95.79	Interaction between SWCNTs and cells/change of cell morphology/
MWCNTs	-	40–60 nm/5–15 µm	-	*E.coli, S. aureus*	removal of rival and bacterial pathogens	38.18, 62.42	Bacterial adhesion or deposition onto bacterial cell
MWCNTs-Ag	Solution mixing	40–60 nm/5–15 µm	-	*E.coli, S. aureus*	removal of rival and bacterial pathogens	86.09, 72.29	Interaction between MWCNTs and cells/change of cell morphology
MWCNTs/lysine, MWCNTs/arginine	Solution mixing	<30 nm/5–15 µm	-	*E.coli, S. aureus, S. typhimurium*	-	-	Electrostatic adsorption on the bacterial cell wall, loss of viability
Fullerene C60	Four step reaction	-	7.5 g/mL/Cyclen-functionalized fullerene	*E.coli, S. aureus*	Antibacterial assay	86.1, 40.7	Electrostatic attraction
Fullerene C70	SES research production	-	2 Wt%/PSP4VP/Ag-NP and polysterene	*E.coli*	Antibacterial assay	5 log	Synergistically target bacterial cells that increase photo-generated ROS
G	low-pressure-CVD	-	AgNW/Water electrolysis	*C. albicans*	Antimicrobial properties	100	Graphene layer reduces the attachment of microbes
GO	Hummers’ method	-	-	*E.coli, S. aureus*	Disinfection activity	-	Mechanism depends on contact time
GO-Ag	Solution mixing	-	-	*E.coli, S. aureus*	Disinfection activity	99.99	ROS depletion of anti-oxidants and protein dysfunction
GO	Hummers’ method	-/0.525 µm	-	*P. aeruginosa*	Antimicrobial properties	92	Oxidative stress, ROS generation, laddering of DNA
rGO	Synthesized from GO	-/3.40 µm	0.1 mg/mL/-	*P. aeruginosa*	Antimicrobial properties	90	Oxidative stress, ROS generation

PVDF: Polyvinylidene fluoride; ROS: Reactive oxygen system; G: Graphene; GO: Graphene oxide; rGO: Reduced graphene oxide; SW-CNTs: Single-walled carbon nanotubes; MWCNTs: Multi-walled carbon nanotubes; CVD: Chemical vapor deposition, 99.99. ^a^ Not mentioned.

## Data Availability

All data generated or analyzed during this study are included in this published article.
